# Liensinine and neferine exert neuroprotective effects via the autophagy pathway in transgenic *Caenorhabditis elegans*

**DOI:** 10.1186/s12906-023-04183-6

**Published:** 2023-10-27

**Authors:** Meng-chen Wu, Ye-hui Gao, Chen Zhang, Bo-tian Ma, Hong-ru Lin, Jin-yun Jiang, Meng-fan Xue, Shan Li, Hong-bing Wang

**Affiliations:** Institute for Regenerative Medicine, Shanghai East Hospital, School of Life Sciences and Technology, Tongji University, Shanghai, 200120 China

**Keywords:** Liensinine, Neferine, Alzheimer’s disease, Neuroprotection, Autophagy

## Abstract

**Background:**

Liensinine and neferine are the main bisbenzylisoquinoline alkaloids obtained from the seeds of *Nelumbo nucifera*, which commonly used as edible food and traditional medicine in Asia. It was reported that liensinine and neferine could inhibit the activities of acetylcholinesterase and cross the blood-brain barriers, suggesting their therapeutic potential for the management of Alzheimer’s disease.

**Methods:**

Here, we employed SH-SY5Y human neuroblastoma cells stably transfected with the human *Swedish* amyloid precursor protein (APP) mutation APP695 (APP695swe SH-SY5Y) as an in vitro model and transgenic *Caenorhabditis elegans* as an in vivo model to investigate the neuroprotective effects and underlying mechanism of liensinine and neferine.

**Results:**

We found that liensinine and neferine could significantly improve the viability and reduce ROS levels in APP695swe SH-SY5Y cells, inhibit β-amyloid and tau-induced toxicity, and enhance stress resistance in nematodes. Moreover, liensinine and neferine had obviously neuroprotective effects by assaying chemotaxis, 5-hydroxytryptamine sensitivity and the integrity of injured neurons in nematodes. Preliminary mechanism studies revealed that liensinine and neferine could upregulate the expression of autophagy related genes (*lgg-1*, *unc-51*, *pha-4*, *atg-9* and *ced-9*) and reduce the accumulation of β-amyloid induced autophagosomes, which suggested autophagy pathway played a key role in neuroprotective effects of these two alkaloids.

**Conclusions:**

Altogether, our findings provided a certain working foundation for the use of liensinine and neferine to treat Alzheimer’s disease based on neuroprotective effects.

**Supplementary Information:**

The online version contains supplementary material available at 10.1186/s12906-023-04183-6.

## Introduction

Alzheimer’s disease (AD), the most common form of dementia, affects more than 50 million people worldwide [1]. With the aggravation of population aging, the incidence of AD is increasing year by year, already inducing tremendous economic and social pressures. However, the pathogenesis remains unclear. There are many reasons for inducing AD, and the cardinal features of Alzheimer’s pathology are amyloid plaques and neurofibrillary tangles (NFTs) [1]. Simultaneously, oxidative stress and autophagy dysfunction also participate in the development of AD by promoting Aβ deposition, tau hyperphosphorylation, and the subsequent loss of synapses and neurons [2]. A crucial role in the suspension of potential damages is the timely drug delivery of neuroprotective medications before AD turns into mildly symptomatic [3].

**Fig. 1:**
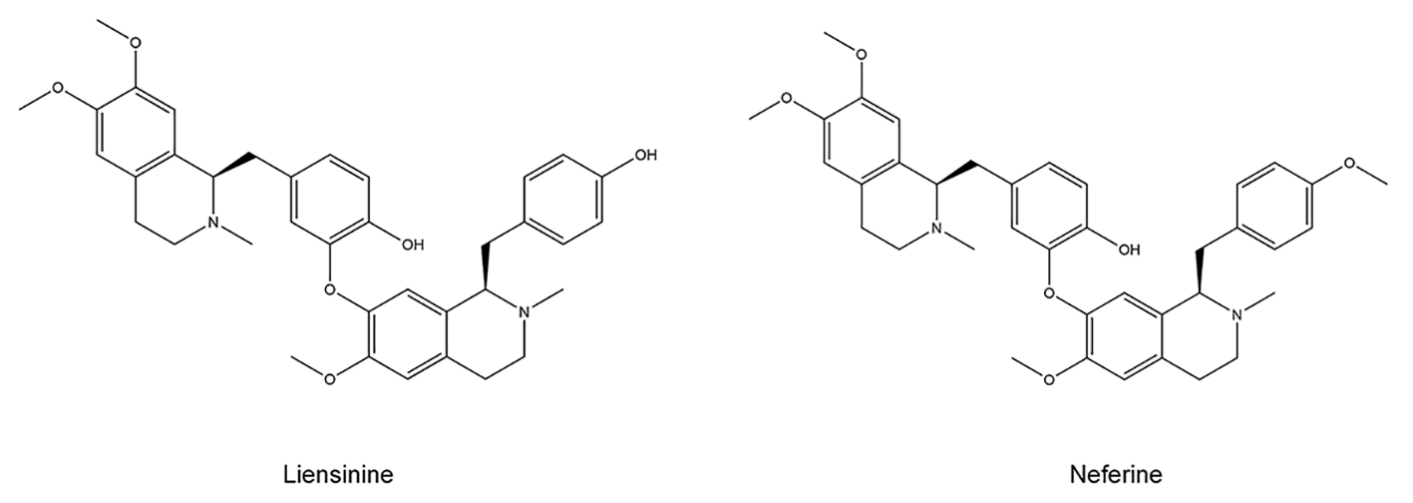
Structure of liensinine and neferine

Alkaloids are small natural molecules that contain nitrogen with significant biological activities and are widely found in nature. Many alkaloids have been reported to have significant neuroprotective effects [4]. ‘Lian Zi Xin’ is the seeds of Lotus (*Nelumbo nucifera*), which is a common traditional Chinese medicine to treat neurodegenerative diseases, insomnia, high fever, and cardiovascular diseases [5]. Liensinine and neferine (Fig. 1) are the main bisbenzylisoquinoline alkaloids of ‘Lian Zi Xin’, and they are known to possess a variety of pharmacological activities including anti-cancer [6–8], antioxidation [9, 10], anti-inflammation [11, 12], treatment of neurodegenerative diseases [13–15] and potential neuroprotective effects [5, 16, 17]. Although there are a few research about the neuroprotective effects of liensinine and neferine, there is a lack of in-depth research on the micro level of neuroprotective effects and treatment of Alzheimer’s disease in cell and *C. elegans* models.

In this paper, we used SH-SY5Y human neuroblastoma cells stably transfected with the human *Swedish* amyloid precursor protein (APP) mutation APP695 (APP695swe SH-SY5Y) as an in vitro model and *Caenorhabditis elegans* (*C. elegans*) as an in vivo model to systematically investigate the neuroprotective effects of liensinine and neferine. APP695swe SH-SY5Y cells are ideal Aβ cell model constructed by transfecting the APP695 *Swedish* mutant gene into SH-SY5Y cells through lentivirus transfection [18]. *C. elegans* is a convenient model in studying neurodegenerative diseases with simple structure, short lifespan and easy cultivation. Although *C. elegans* doesn’t have Aβ and tau genes, it can be easily constructed by gene editing to express human Aβ and tau protein in their muscle cells and neurons for screening AD-related drugs and clarifying their mechanisms [19].

## Materials and methods

### Preparation of liensinine and neferine

Liensinine and neferine (Batch No. 10,532 and 9813, respectively) were purchased from Shanghai Standard Technology Company (China). Due to the insolubility of liensinine and neferine in water, it is necessary to use DMSO for dissolution, while ensuring that the concentration of DMSO is below 0.3% in *C. elegans* and 0.1% in cell. Hence, we need to prepare a stock solution of 333 mM and dilute it with s-complete buffer to achieve a concentration of 10 mM. Subsequently, we dilute the 10 mM solution to the corresponding working solution.

### Cell cultures and treatments

SH-SY5Y cells were purchased from American Type Culture Collection (ATCC), cultured in Minimum Essential Medium (MEM) and F12 (1:1) (Thermo Fisher Scientific, America) supplemented with 10% Fetal Bovine Serum (FBS) (Gibco, America) and 1% Penicillin-Streptomycin Solution (PS) (Gibco, America) at 37 °C, in a 5% CO_2_ atmosphere. APP695swe SH-SY5Y cells stably transfected with the human *Swedish* mutation were generated as previously described [20], human APP *Swedish* mutant gene (APP695swe) was transfected into SH-SY5Y cells by lentiviral transfection to induce the expression of Aβ protein. One day prior to transfection, cells were seeded onto 6-well plates and 500 µL of pre-packaged target plasmid and control plasmid virus solution were added. After 24 h, the regular culture medium was replaced. Successful transfection could be observed under a microscope after 48–72 h, and stable transgenic plants can be identified based on their resistance markers.

### Cell viability assay

4,5-dimethyl-2-thiazolyl-2,5-diphenyl-2-H-tetrazom bromide (MTT) (Nanjing Jiancheng Biological Engineering Research Institute) assay was used to study the effect of liensinine and neferine on cell viability. A total of 100 µL suspensions (1 × 10^3^ cells/well) were planted onto 96-well plates overnight and the treated with different doses of liensinine and neferine for 24 h, which was dissolved in 0.1% DMSO. Added 50 µL MTT solution into each well, and cultured for 4 h. Carefully sucked the culture medium, and added 150 µL DMSO to fully dissolve the crystals. The absorbance value was measured at OD570 of enzyme-linked immunosorbent assay.

### Determination of ROS levels

The ROS level of APP695swe SH-SY5Y cells was measured using the human reactive oxygen species (ROS) ELISA Kit (Shanghai Enzyme-linked Biotechnology). Collected samples and removed particulates by centrifugation for 10 min at 3000×g. Set standard wells and testing wells, added different concentrations of standard 50 µL to the standard wells;added testing sample 10 µL and then added sample diluent 40 µL to testing sample wells. Added 100 µL HRP-conjugate reagent to each well and incubated for 60 min at 37 °C. Added 50 µL chromogen solution A and 50 µL chromogen solution B to each well, gently mixed, and incubated for 15 min at 37 °C protected from light. Finally, added 50 µL stop solution to each well and read the Optical Density (O.D.) at 450 nm.

### Strains and maintenance conditions

*Caenorhabditis elegans* strains used in this study were obtained from the Caenorhabditis Genetics Center (CGC), University of Minnesota (Minneapolis, MN, USA), and the Sunybiotech company. N2 (wild-type), CL4176: dvIs27 [Pmyo-3::A-Beta(1–42)::let-851 3’UTR + rol-6(su1006)], CL2355: dvIs50 [pCL45snb-1::A-Beta(1–42)::3’UTR(long) + mtl-2::GFP], GMC101: dvIs100 [unc-54p::A-beta-1-42::unc-54 3’-UTR + mtl-2p::GFP], BR5706: bkIs10 [aex-3p::hTau V337M + myo-2p::GFP] and VH254: hdEx81 [F25B3.3::tau352(PHP) + pha-1(+)] were obtained from the Caenorhabditis Genetics Center (CGC), University of Minnesota (Minneapolis, MN, USA). PHX3692 [unc-47p::mcherry + pCL45snb-1::A-Beta(1–42)::3’UTR(long) + mtl-2::GFP] and PHX3392 [lgg-1::mCherry::GFP:lgg-1 + unc-54p::A-beta-1-42::unc-54 3’-UTR + mtl-2p::GFP] were constructed by the Sunybiotech company. All strains were cultured at 20 °C on solid nematode growth medium (NGM) plates with *Escherichia coli* (E. *coli*) OP50 except CL4176, CL2355, and PHX3692 that were cultured at 16 °C.

### Aβ induced paralysis assay

Aβ induced paralysis assay was performed as described previously [21], synchronized Aβ transgenic CL4176 nematodes were maintained on an 35 mm NGM culture plate containing *E. coli* OP50, liensinine and neferine. Cultured at 16 °C for 36 h and then raised the temperature to 23 °C for Aβ expression. Scored the paralyzed nematodes began at 24 h after raising the temperature. If the nematodes can’t move normally only their heads shake slightly or there is a transparent circular sterile circle around the head, they are considered paralyzed. Recorded the number of paralyzed nematodes per hour until all of them in the culture plate were paralyzed. The assay was repeated at least three times and calculated the PT_50_ (the time when 50% nematode paralysis occurs - the time when the control group begins paralysis).

### Chemotaxis assay

Chemotaxis paralysis assay was performed as described previously [22], 1 M Sodium acetate (Sangon Biotech, China) and 1 M Sodium azide (Sangon Biotech, China) (1:1) were blended as attractants. 1 M sterile water and 1 M Sodium acetate (1:1) were blended as the control odorant. Synchronized L1 CL2355 nematodes were maintained on a 100 mm NGM culture plate with *E. coli* OP50, liensinine and neferine. Cultured at 16 °C for 42 h and then raised temperature to 25 °C for 32 h. Washed the nematodes with M9 buffer to remove the *E. coli* OP50 and drugs, and transferred the nematodes to the center of an uncoated 100 mm NGM plate. Dripped 10 µL attractant and 10 µL control odorant on both sides of the plate respectively, placed upside down at room temperature for one hour. Calculated the chemotaxis index (CI) = (number of nematodes on the attractant side − number of nematodes on the control side)/total number of nematodes.

### 5-hydroxytryptamine sensitivity assay

For 5-hydroxytryptamine sensitivity assay, synchronized L1 CL2355 nematodes were maintained on a 35 mm NGM culture plate with *E. coli* OP50, liensinine and neferine. Cultured at 16 °C for 42 h and then raised temperature to 25 °C for 32 h. Washed the nematodes with M9 buffer to remove the *E. coli* OP50 and drugs, and transferred to a 96-well plate with 150 µL 5 mg/ml 5-hydroxytryptamine (Sangon Biotech, China). Continued to incubate at 25 °C for 12 h and recorded the number of active nematodes.

### Neuronal integrity assay

Under normal conditions, the dorsal nerve cord and abdominal nerve cord and the neurons connecting the middle of the two cords can be observed [23]. Due to the expression of Aβ, the neuronal integrity of the PHX3692 nematodes has been seriously affected. Synchronized L1 PHX3692 nematodes were maintained on a 35 mm NGM culture plate with *E. coli* OP50, liensinine and neferine. Cultured at 16 °C for 42 h and then raised the temperature to 25 °C for 32 h to induce Aβ expression. Dropped 5 µL 90% oil onto the prepared 2% agarose pad and fixed the treated nematodes on the agarose pad with a sealing agent, as described previously [24]. The neurons of nematodes can be observed by a fluorescence microscope (Revolve FL, Echo Laboratories, USA). Counted the number of D-type motor neurons in the abdominal nerve cord.

### Movement distance assay

To detect the effect of liensinine and neferine on the motion ability of nematodes, synchronized L1 BR5706 nematodes were cultured in a 96-well plate with *E. coli* OP50, 1.08 mM FUDR was added in L3 and after 24 h liensinine and neferine were added. Washed the nematodes with M9 buffer to remove the *E. coli* OP50 and drugs, transferred to an uncoated 35 mm NGM plate. Used WormLab software to record the Track Length (µm) [forward + reversals] and Track duration (s), deleted data with a time less than 10s, and then calculate the movement distance within 10s.

### Whipping rate assay

To detect the effect of liensinine and neferine on the whipping rate of nematodes, synchronized L1 VH254 nematodes were cultured in a 96-well plate with *E. coli* OP50, 1.08 mM FUDR was added in L3 and after 24 h liensinine and neferine were added. Washed the nematodes with M9 buffer to remove the *E. coli* OP50 and drugs. Calculated the whipping rate in the liquid within 10 s after treatment with liensinine and neferine on day 4.

### Stress resistance assay

For the oxidative stress assay in vitro, synchronized L1 wild-type nematodes were cultured in a 96-well plate with *E. coli* OP50, 1.08 mM FUDR was added in L3 and after 24 h liensinine and neferine were added. Washed the nematodes with M9 buffer to remove the *E. coli* OP50 and drugs, transferred to a transparent 96-well plate with 50 mM paraquat (Sigma, Germany), and recorded the alive nematodes every 12 h until all died.

### Measurement of reactive oxygen species (ROS)

Endogenous reactive oxygen species (ROS) levels were measured using fluorescent probe 2′,7′-dichlorodihydrofluorescein diacetate (H_2_DCF-DA) (Sigma, Germany), as described previously [25]. The nematodes were treated as in the paralysis assay, washed the nematodes with M9 buffer to remove the *E. coli* OP50 and drugs. Picked nematodes from the same period and transferred to a black bottom 96-well plate with 1 mM fluorescent probe 2′,7′-dichlorodihydrofluorescein diacetate (H_2_DCF-DA). Avoided light and incubated at 37 °C for 2 h, measured with a fluorescence microplate reader (excitation wavelength: 485 nm; emission wavelength: 535 nm).

### Total RNA isolated and RT-PCR

The nematodes were treated as in the paralysis assay, ensuring that the total count of nematodes was no less than 3000. Washed the nematodes with M9 buffer to remove the *E. coli* OP50 and drugs, and then collected in s-complete buffer at -80℃ for 2 h to facilitate cracking. Total RNA was isolated by Trizol (Tiangen, China) and converted to cDNA with an All-in-One cDNA synthesis supermix kit by Quantitative Reverse Transcription PCR. Quantitative real-time PCR (RT-PCR) was performed using a 2 × SYBR Green qPCR master mix kit (Bimake, China) and a LightCycler96 Real-time PCR system (Roche, Switzerland) to measure mRNA expression levels. Actin was used as a reference gene, and data were analyzed by the 2^−ΔΔCt^ method.

### Fluorescence assay

The autophagosome accumulation PHX3392 model was constructed by Aβ transgenic GMC101 nematodes to observe the effect of liensinine and neferine on autophagosome accumulation [26]. Synchronized PHX3392 nematodes were maintained on a 35 mm NGM culture plate with *E. coli* OP50, liensinine and neferine. Cultured at 20 °C for 42 h and then raised the temperature to 25 °C for 8 h. Dropped 5 µL M9 buffer onto the prepared 2% agarose pad, and fixed the treated nematodes on the agarose pad with a sealing agent, as described previously [27, 28]. Observed the fluorescence using the fluorescence microscope (Revolve FL, Echo Laboratories, USA).

### Western blotting

The PHX3392 nematodes were treated as in the fluorescence assay, ensuring that the total count of nematodes was no less than 2000. Washed the nematodes with M9 buffer to remove the *E. coli* OP50 and drugs, and then collected in 1x phosphate buffer solution at -80℃ for 2 h to facilitate cracking. Total protein was extracted with ice-cold radioimmunoprecipitation assay (RIPA) lysis buffer (Beyotime Biotechnology, China) containing 1x protease inhibitor and 1x phosphatase inhibitor cocktail (Beyotime Biotechnology, China) and loading buffer was added after protein quantification using BCA kit (Thermo Fisher Scientific, America) to prevent protein degradation. A 10–180 kDa protein maker (Solarbio, China) was used as an indicator of molecular weight. Protein samples were run at 30 V for 40 min on a stacking gel, and at 80 V for 120 min on a separating gel. The mCherry-GFP (green flurescent protein) -LGG-1 levels were detected with rabbit anti-GFP monoclonal antibody (dilution 1:1000, Cell Signaling Technology), β-actin monoclonal antibody (dilution 1:1000, Proteintech) was used as the internal control. Anti-mouse IgG HRP-linked antibody (dilution 1:2000, Cell Signaling Technology) was the secondary antibody. Protein bands were detected using the standard enhanced chemiluminescence (ECL) western blotting Substrate. Mean densities of the mCherry-GFP-LGG-1 bands were analyzed using Image J.

### Statistical analysis

Each assay was repeated at least three times. We used GraphPad Prism 8.0 and Image J to analyze the data. Student’s *t-*test was used to compare two groups and One-way analysis of variance (ANOVA) was used to compare multiple groups. *P* < 0.05 was considered statistically significant.

## Results

### Liensinine and neferine improved APP695swe SH-SY5Y cell viability

**Fig. 2:**
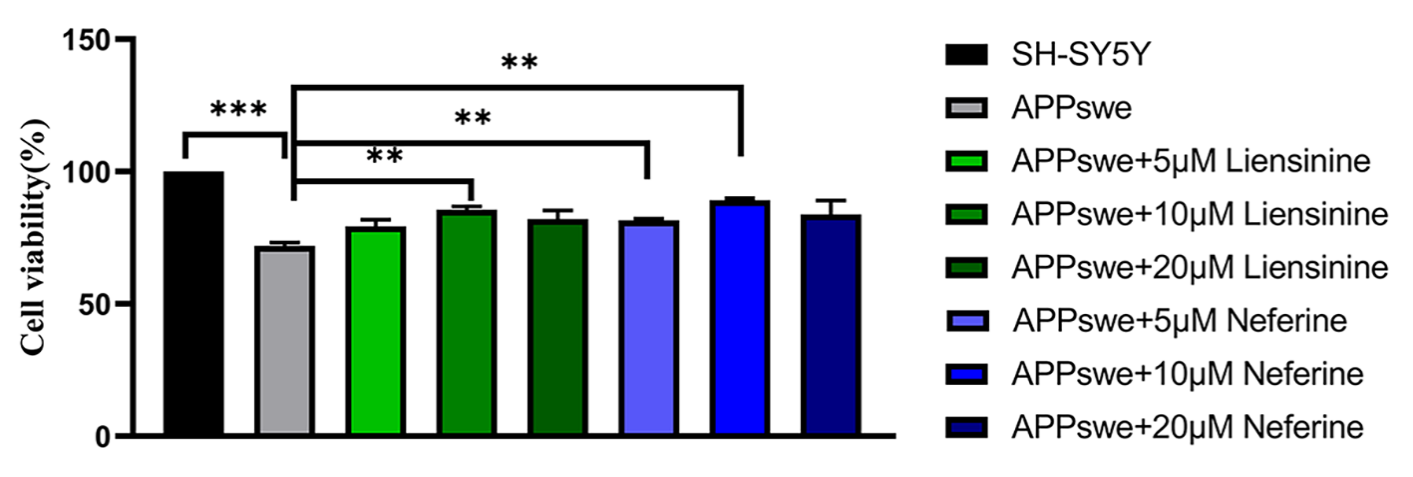
Liensinine and neferine improved APP695swe SH-SY5Y cell viability. Cell viability of APP695swe SH-SY5Y cells at various liensinine and neferine concentrations (5, 10, 20µM). (n = 3, **p < 0.01; ***p < 0.001)

To explore the effects of liensinine and neferine on intracellular Aβ toxicity, we used Aβ-induced neurotoxicity in APP695swe SH-SY5Y cells, which constructed by stably transfecting with the human *Swedish* APP mutation APP695 in SH-SY5Y human neuroblastoma cells, as in vitro Aβ cell model. APP695swe SH-SY5Y cells was treated with liensinine and neferine at concentrations of 5, 10 and 20 µM respectively. We found that both liensinine and neferine could improve cell viability, and 10 µM liensinine and neferine showed the most significant effect, increased by 18.90% and 23.91% compared with APP695swe blank control (Fig. 2), indicating that liensinine and neferine could significantly inhibit intracellular Aβ toxicity.

### Liensinine and neferine decreased ROS levels in APP695swe SH-SY5Y cells

**Fig. 3:**
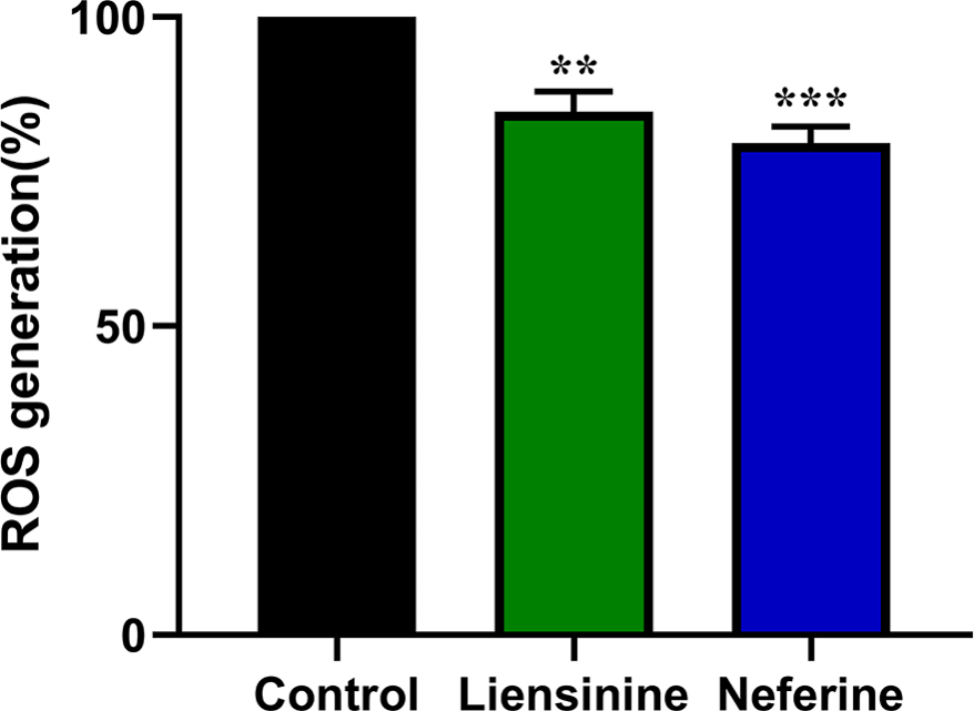
Liensinine and neferine reduced intracellular levels of ROS. Liensinine and neferine reduced intracellular ROS levels in APP695swe SH-SY5Y cells. (n = 3, **p < 0.01; ***p < 0.001)

Oxidative stress plays a critical role in AD development, and it can even be considered a crucial central factor in the pathogenesis of AD [29]. We used APP695swe SH-SY5Y cells to explore whether liensinine and neferine could enhance oxidative stress resistance. After being treated with 10 µM liensinine and neferine, the intracellular ROS levels in treated APP695swe SH-SY5Y cells decreased by 15.31% and 20.37% compared with untreated group (Fig. 3), which suggested that liensinine and neferine could improve cellular antioxidant capacity.

### Liensinine and neferine delayed Aβ-induced toxicity in C. elegans

**Fig. 4:**
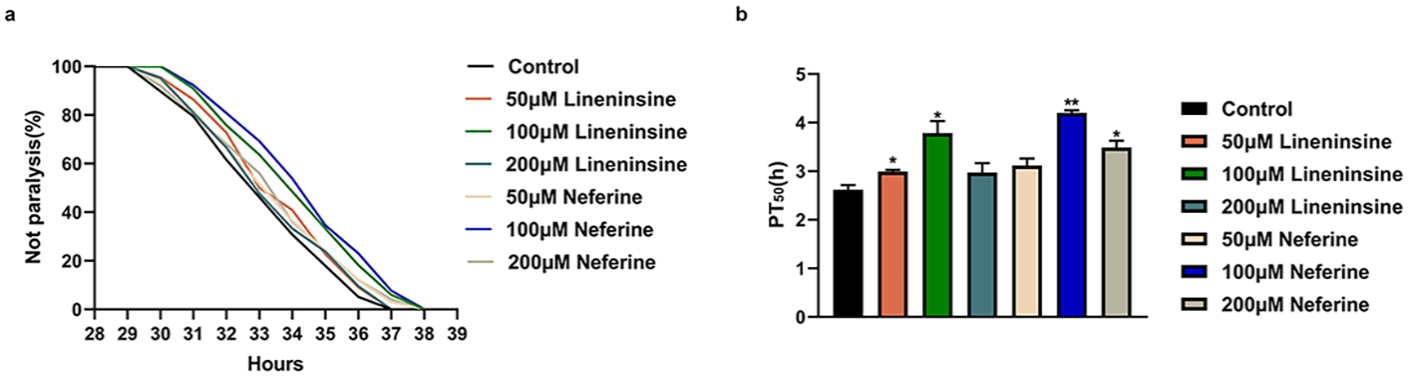
Liensinine and neferine delayed Aβ-induced toxicity in *C. elegans*. (**a**) Percentage of nematodes not paralyzed at various liensinine and neferine concentrations (50,100,200µM). (**b**) The PT50 was used for quantitative analysis, which represents the time interval from the beginning of paralysis to 50% of nematode paralysis. (n = 3, *p < 0.05; **p < 0.01) Each group contains more than 30 nematodes

Many lines of evidence support that β-amyloid (Aβ) peptides play an important role in AD, the most common cause of dementia. Reduction in Aβ has been the major target of recent experimental therapies against AD [30]. To explore the effects of liensinine and neferine on Aβ toxicity in nematodes, we used the transgenic CL4176 nematodes expressing temperature-induced human Aβ protein in the muscle cells. With the increase of Aβ expression, CL4176 showed a gradual paralysis phenotype. CL4176 nematodes were treated with liensinine and neferine at 50, 100 and 200 µM concentrations respectively. The results showed that nematodes exposed to 100µM liensinine and neferine had the most significant effect on prolonging mean paralysis time, which was 44.66% and 60.31% longer than the control (Fig. 4), indicating that liensinine and neferine could inhibit Aβ-induced toxicity to nematodes. Consequently, we used 100 µM liensinine and neferine as the treatment concentration for subsequent experiments.

### Liensinine and neferine had protective effects on Aβ-induced neuronal damage in C. elegans

A characteristic of AD is the extracellular deposition of β-amyloid (Aβ) plaques, which is thought to provoke neuroinflammation, neuronal dysfunction, and progressive neuronal loss [31]. To explore the protective effects of liensinine and neferine on Aβ-induced neuronal damage, we firstly used the transgenic strain CL2355, which *pan*-neuron expressed Aβ, to measure two characteristic neuronal controlled behaviors, chemotaxis and 5-hydroxytryptamine sensitivity.

**Fig. 5:**
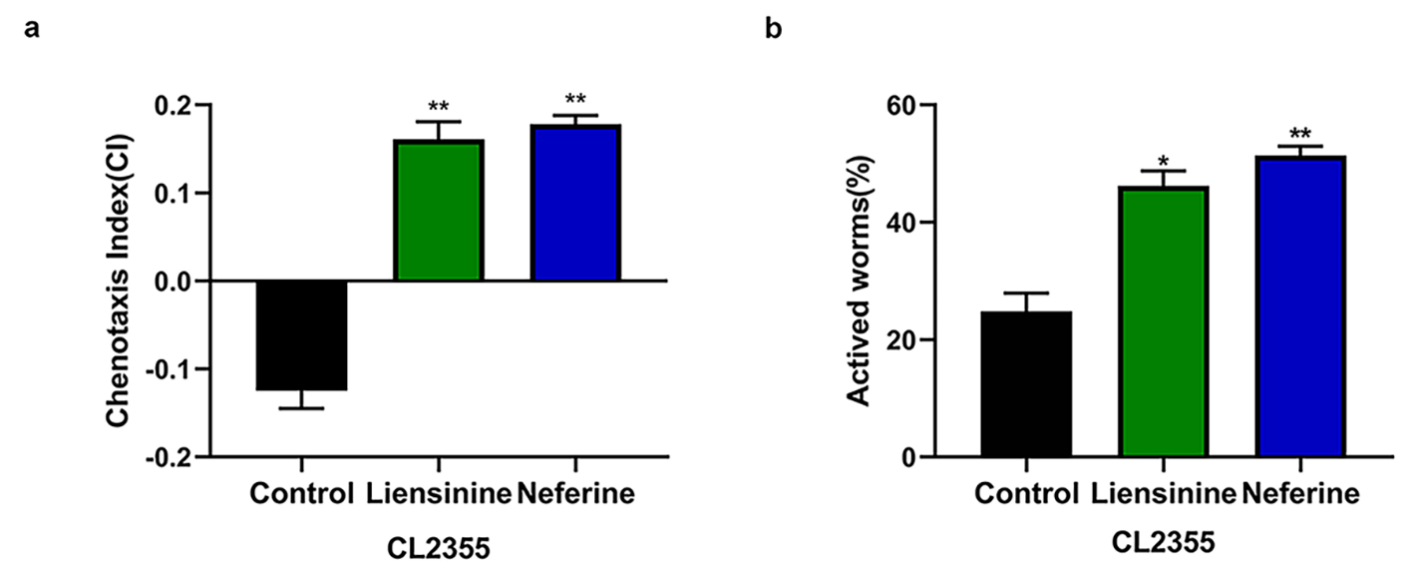
Liensinine and neferine enhanced chemotaxis and 5-hydroxytryptamine sensitivity in *C. elegans*. (**a**) Liensinine and neferine increased the Chemotoxis Index (CI) of CL2355 nematodes expressing neuronal Aβ. (**b**) The proportion of active CL2355 nematodes after being treated with liensinine and neferine. (n = 3, *p < 0.05; **p < 0.01) Each group contains more than 30 nematodes

The chemotaxis response in *C. elegans* is mediated by the activation of several sensory neurons and interneurons to stimulate the motor neurons [32]. 5-hydroxytryptamine is a key neurotransmitter that modulates several behaviors of *C. elegans*, including egg-laying, locomotion, and olfactory learning [33]. When exogenous 5-hydroxytryptamine was applied to the nematodes, they become paralyzed as a result of the sensitivity to excessive 5-hydroxytryptamine. Compared to the control group, 100 µM liensinine and neferine could significantly improve the chemotaxis index (CI) value and vitality of nematodes respectively (Fig. 5), which suggested 100µM liensinine and neferine could effectively protect Aβ-induced neuronal damage in *C. elegans*.

**Fig. 6:**
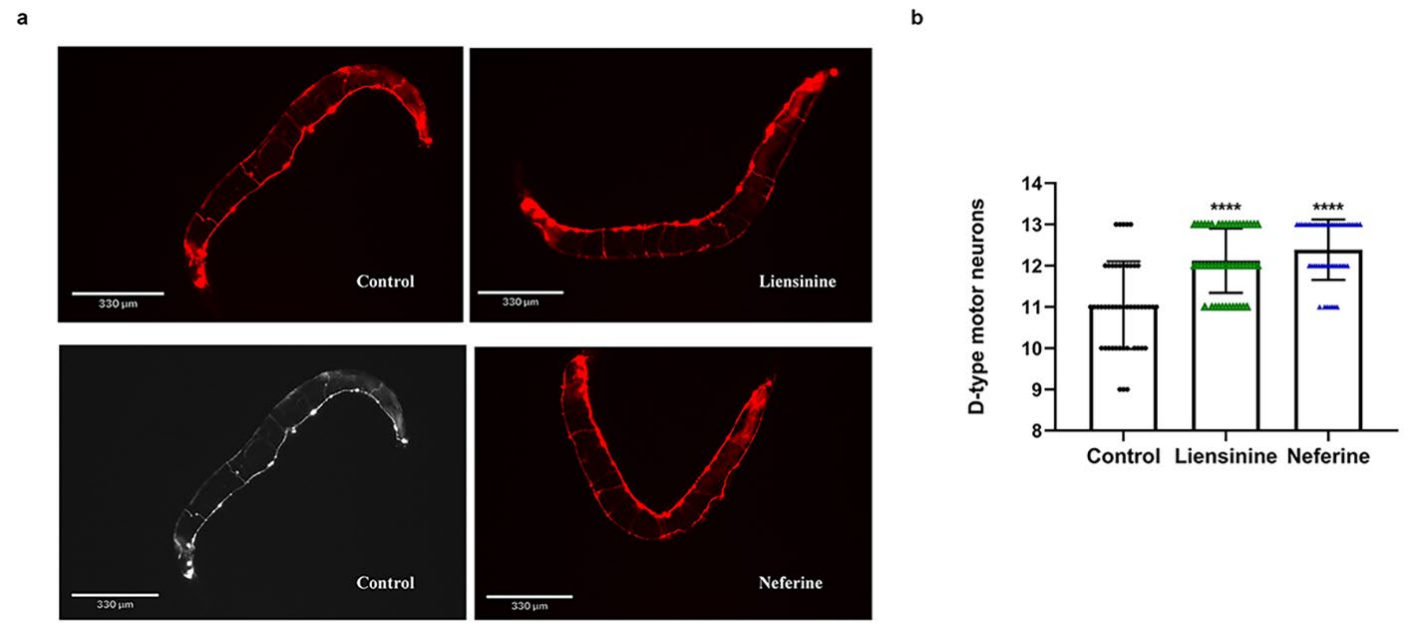
Liensinine and neferine protected neuronal integrity in *C. elegans*. (**a**) D-type motor neurons of PHX3692 nematodes shown by fluorescence confocal microscope. (**b**) The number of D-type motor neurons after being treated with liensinine and neferine. (n = 3, ****p < 0.0001) Each group contains more than 30 nematodes

The neurotransmitter γ-aminobutyric acid (GABA) is an inhibitory synapse and is important in *C. elegans* motor neurons. 26 of the 302 neurons present in *C. elegans* express the neurotransmitter GABA. In the ventral nerve cord, 6 DD-like and 13 VD-like cells express GABA [23], with the expression of Aβ, it is commonly to cause neuronal damage and loss. Therefore, we furtherly used the transgenic PHX3692 nematodes, D-type motor neurons marked with mCherry, to assess the neuroprotective effects of liensinine and neferine. As all nematodes possess three D-type neurons in the head and one in the tail, we solely identified 15 mid-body D-type neurons. Due to the expression of Aβ, the number of D-type neurons of PHX3692 nematodes is seriously decreased. After being treated with 100 µM liensinine and neferine respectively, the number of D-type motor neurons in PHX3692 strains was obviously increased compared with control (Fig. 6). According to above experiments, it was strongly suggested that liensinine and neferine showed a significant protective effect on nerve damage induced by Aβ.

### Liensinine and neferine inhibited the expression of hyperphosphorylated tau protein

**Fig. 7:**
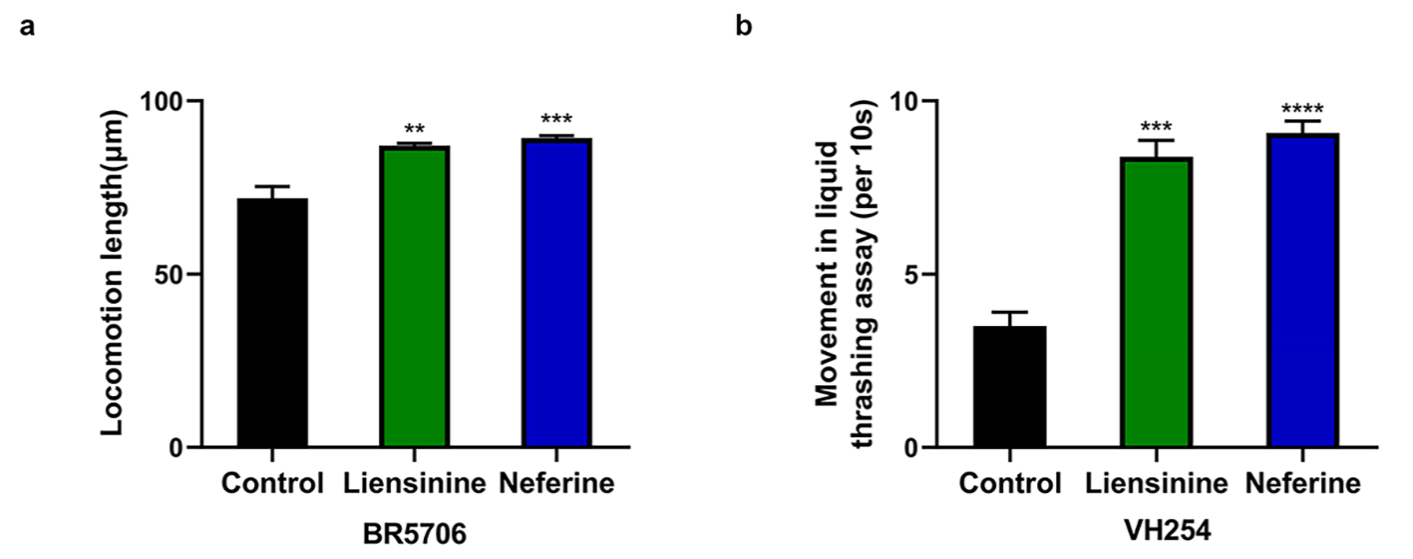
Liensinine and neferine inhibited the expression of hyperphosphorylated tau protein in *C. elegans*. (**a**) Liensinine and neferine improved motion ability of BR5706 nematodes. (**b**) Liensinine and neferine improved the whipping rate of VH254 nematodes. (n = 3, **p < 0.01; ***p < 0.001; ****p < 0.0001) Each group contains more than 30 nematodes

Accumulation of phosphorylated tau is a key pathological feature of AD. Phosphorylated tau accumulation causes synaptic impairment, neuronal dysfunction, and neurofibrillary tangles formation [34]. To investigate the inhibitory effects of liensinine and neferine on hyperphosphorylated tau protein, we first used BR5706 nematodes, which have severe movement defects due to excessive phosphorylation of tau protein in the body to detect the motion ability. The results showed that 100 µM liensinine and neferine could significantly improve motion vitality of nematodes respectively (Fig. 7A), which suggested that liensinine and neferine had protective effects on motion damage caused by abnormal phosphorylated tau protein expression.

To further investigate the effects of liensinine and neferine on damage caused by abnormal phosphorylated tau protein expression in *C. elegans*, we used VH254 nematodes, which became increasingly uncoordinated movement with age due to hyperphosphorylation of tau protein in the body. After being treated with 100 µM liensinine and neferine respectively, the whipping rate was effectively improved (Fig. 7B). It was further confirmed that liensinine and neferine had protective effects on motion damage caused by abnormal phosphorylated tau protein expression.

### Liensinine and neferine improved antioxidative stress resistance in C. elegans

Oxidative stress occurs due to a disparity in redox states brought on by either an excessive generation of reactive oxygen species (ROS) or a reduction in antioxidant function [35]. Evidence has suggested that oxidative stress may augment the production and aggregation of Aβ and facilitate the phosphorylation and polymerization of tau, thus forming a vicious cycle that promotes the initiation and progression of AD [36].

**Fig. 8:**
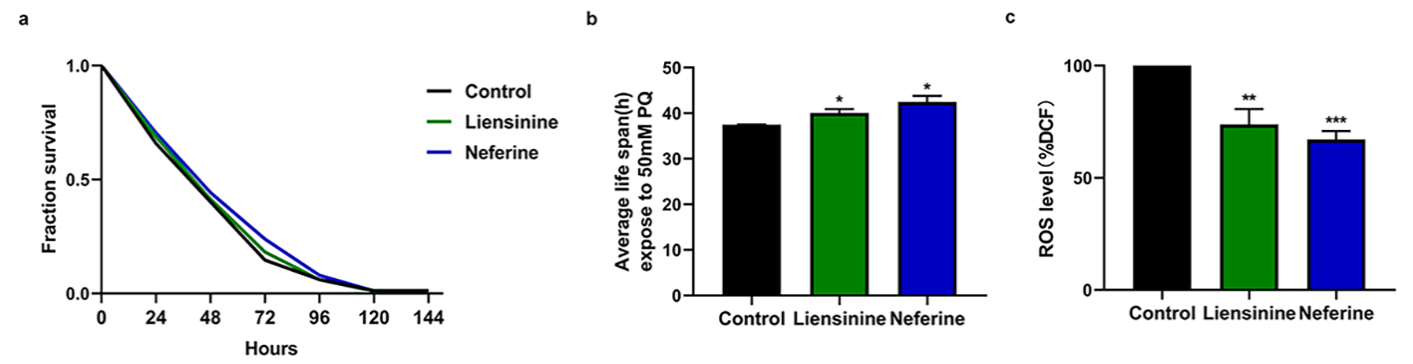
Liensinine and neferine enhanced oxidative stress resistance and decreased the accumulation of ROS in *C. elegans*. (**a-b**) Liensinine and neferine enhanced the number of surviving nematodes exposed to 50mM paraquat. (**c**) Liensinine and neferine decreased intracellular ROS levels measured by fluorescent prob H_2_DCF-DA. (n = 3, *p < 0.05; **p < 0.01; ***p < 0.001) Each group contains more than 30 nematode

**Fig. 9:**
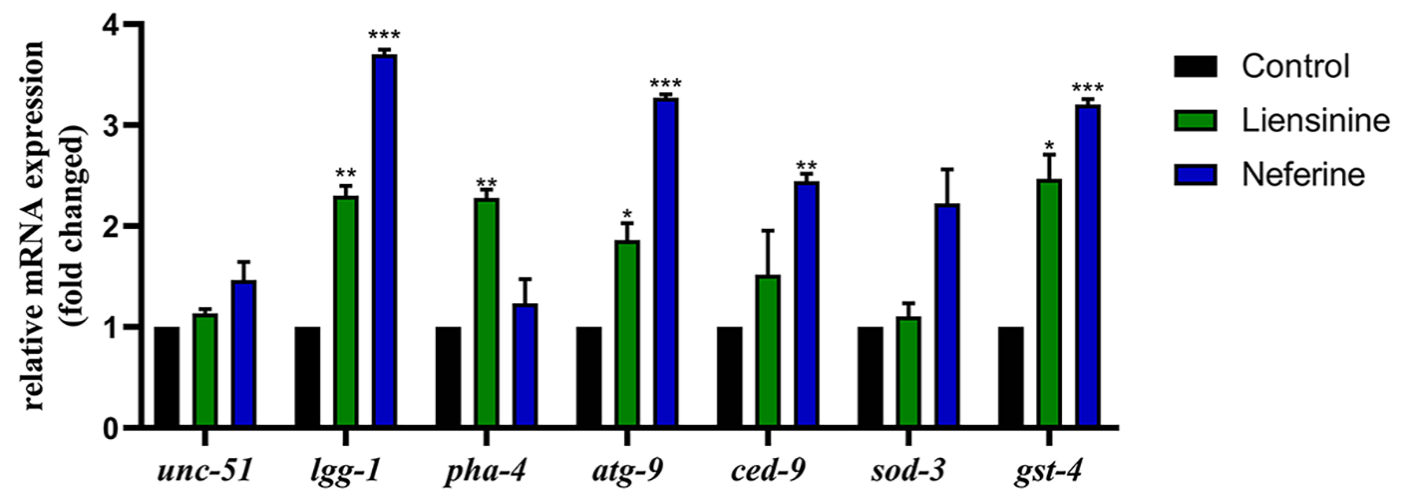
Liensinine and neferine exerted a neuroprotective effect through the autophagy pathway. Gene expression levels after being treated with liensinine and neferine. (n = 3, *p < 0.05; **p < 0.01; ***p < 0.001) Each group contains more than 2000

When exogenous 50 mM paraquat is applied to wild-type nematodes (N2), it causes acute oxidative stress injury. We used it to explore whether liensinine and neferine could enhance oxidative stress resistance of nematodes. We found that 100 µM liensinine and neferine respectively prolonged the average lifespan of N2 worms by 6.94% and 13.40% compared to the control (Fig. 8A and B). To evaluate the impact of liensinine and neferine to intracellular ROS levels, we measured the ROS accumulation at 32 h after up shifting the temperature using H_2_DCF-DA (a fluorescent probe that reacts with ROS). It was observed that the intracellular ROS level in nematodes was obviously decreased by 26.23% and 32.87% compared to the control (Fig. 8C). Additionally, both liensinine and neferine could upregulate the mRNA levels of downstream key genes, *sod-3* and *gst-4*, of oxidative stress (Fig. 9). Combined with above experiments, all results demonstrated that liensinine and neferine could significantly enhance oxidative stress resistance of nematodes.

### Liensinine and neferine upregulated the expression of autophagy related genes

Autophagy is an essential degradation pathway in clearing abnormal protein aggregates in mammalian cells and is responsible for protein homeostasis and neuronal health [37]. Autophagy-lysosome defects occur early in the pathogenesis of AD and have been proposed to be a significant contributor to the disease process [38]. The accumulation of Aβ and tau could inhibit the autophagosome formation, another, abnormal fusion of autophagosomes and lysosomes was observed with accumulation of misfolded proteins, which induced the abnormal accumulation of autophagosomes [39]. The phenomenon of autophagosome accumulation has been observed in brains of AD patient [40].

To clarify the underlying mechanism of neuroprotective effects of liensinine and neferine, firstly, we assayed the mRNA levels of autophagy membrane formation key genes, *lgg-1*, *unc-51*, and *atg-9*. The results showed that both liensinine and neferine could obviously increase the expression of *lgg-1*, *atg-9* (Fig. 9), which suggested that liensinine and neferine could enhance the autophagosome formation, and active autophagy pathway to exert neuroprotective effects. Furtherly, we assayed the mRNA levels of autophagy process key genes, *pha-4* and *ced-9*. The results showed that liensinine could obviously increase the expression of *pha-4*, while neferine could obviously increase the expression of *ced-9* (Fig. 9).

**Fig. 10:**
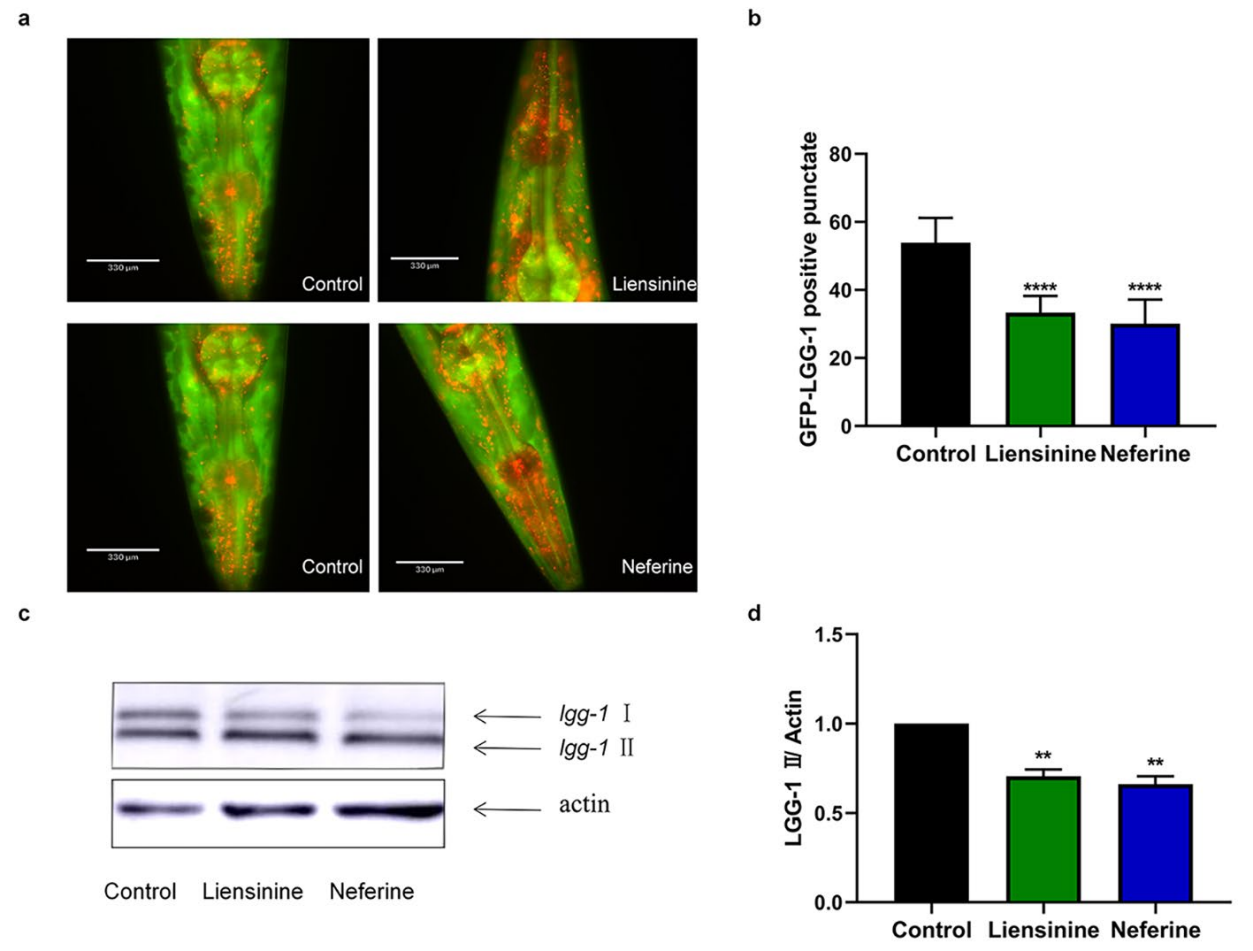
Liensinine and neferine reduced accumulation of autophagosomes. (**a**) The fluorescence diagram of autophagosomes and autolysosomes after being treated with liensinine and neferine. (**b**) The number of autophagosomes after being treated with liensinine and neferine. (**c**) The western blot of LGG-1-I/II. (**d**) Quantified western blot of gel densities by Image J. (n = 3; **p < 0.01; ****p < 0.0001) Each group contains the same number of nematodes

Moreover, we conducted an autophagosome accumulation assay in PHX3392 nematodes, which autophagosomes were accumulated and visualized as GFP-positive punctate. The results showed that 100 µM liensinine and neferine could significantly reduce the accumulation of autophagosomes in nematodes (Fig. 10A and B). LGG-1-II is a membrane protein on the autophagosome of nematodes, and its protein expression level can indirectly represent the number of autophagosomes. After treatment with 100 µM liensinine and neferine respectively, we observed that the expression level of LGG-1-II protein in PHX3392 nematodes was obviously decreased (Fig. 10C and D), furtherly confirmed liensinine and neferine could reduce the accumulation of autophagosomes.

Above experiments suggested that liensinine and neferine promote the formation of autophagosomes while reducing their abnormal accumulation, which may be related to activating the fusion of autophagosomes and lysosomes.

## Discussion

Natural small molecules of alkaloids extracted from plants have been a long way for human beings to fight against numerous diseases. In the folk, ‘Lian Zi Xin’, the embryo of *Nelumbo nucifera*, is often used to make functional and healthcare foods, and is considered conducive to the prevention of AD [41]. Liensinine and neferine are main alkaloid constituents isolated from *Nelumbo nucifera* [42]. It was reported that liensinine and neferine have the protective effects on Aβ25–35 -injured PC12 cells [16], and total alkaloids (mainly contains liensinine, isoliensinine, and neferine) from *Nelumbo nucifera* could improve cognitive impairment in APP/PS1 mice and protect Aβ-damaged PC12 cells [17]. In this paper, we furtherly demonstrated their neuroprotective effects and clarified their underlying mechanism by using APP695swe SH-SY5Y cells as in vitro model and *C. elegans as in vivo* model.

The occurrence of Alzheimer’s disease is related to many factors, the two core pathological hallmarks are amyloid plaques and neurofibrillary tangles formed by hyperphosphorylated tau protein. The amyloid cascade hypothesis suggests that the deposition of β-amyloid (Aβ) triggers neuronal dysfunction and death in the brain [43]. According to our results, liensinine and neferine could significantly inhibit intracellular Aβ toxicity (Fig. 2), and obviously ameliorate Aβ toxicity, protect the integrity of neurons and improve the chemotaxis and 5-hydroxytryptamine sensitivity in nematodes (Figs. 4, 5 and 6), the effect of neferine is more significant than that of liensinine. The above results furtherly confirmed that liensinine and neferine could inhibit the toxicity of Aβ protein and have a protective effect on neuronal damage induced by Aβ protein.

In tauopathies, tau protein becomes hyperphosphorylated, detaches from microtubules, abnormally localizes to the soma and dendrites, is cleaved by caspases and aggregates into neurofibrillary pathology. These processes disrupt cellular transport and cause synapse loss and, ultimately, many neurons die causing disrupted neural circuits and cognitive decline [44]. Human tau in *C. elegans* is highly phosphorylated at many disease-relevant sites and exhibits conformational changes similar to tau in AD. Both, wild-type tau- and PHP tau-expressing animals develop a phenotype of uncoordinated locomotion. However, only worms expressing PHP tau show a defective pattern of motor neuron [45]. The movement distance and whipping rate are two parameters employed to measure the motility of nematodes. According to our results, liensinine and neferine could significantly improve motion ability and whipping rate of nematodes (Fig. 7), which suggests that liensinine and neferine had protective effects on motion damage caused by abnormal phosphorylated tau protein expression.

Oxidative stress participates in the development of AD by promoting Aβ deposition, tau hyperphosphorylation, and the subsequent loss of synapses and neurons [2]. Thus, we evaluated the impact of liensinine and neferine on APP695swe cells and nematodes antioxidant capacity. We found that liensinine and neferine could decrease intracellular ROS levels, resist paraquat-induced oxidative stress and reduce the accumulation of ROS in nematodes (Fig. 8), which suggests that liensinine and neferien could reduce oxidative damage and resist Aβ-caused damage.

Liensinine and neferine are very similar in structure, with only one methyl difference (Fig. 1). The introduction of methyl groups can effectively improve the pharmacological effects of molecules, including improving the physical and chemical properties of drugs, enhancing the interaction between small molecules and target proteins, and improving the metabolic properties of drugs [46]. It can also be seen from our experimental results that the effect of neferine is more significant than that of liensinine.

Autophagy is an evolutionarily conserved lysosome-mediated degradation process and is essential for survival, development, and organismal homeostasis. Emerging facts showed that defect in autophagy were likely to contribute to AD. Autophagy-lysosome defects occur early in the pathogenesis of AD and have been proposed to be a significant contributor to the disease process [38]. According to the results, we found that both liensinine and neferine could upregulate the mRNA levels of autophagosome formation and autophagy process key genes (Fig. 9), indicating that liensinine and neferine could enhance autophagy activity. Additionally, the impairment in the autophagy-lysosome system disturbs the turnover of other molecules associated with AD, which may also contribute to neuronal dysfunction in AD [47]. Generally, during autophagy, autophagosomes fuse with lysosomes to form autolysosomes to degrade misfolded proteins. Abnormal fusion of autophagosomes and lysosomes can lead to the abnormal accumulation of autophagosomes [40], which has been observed in brains of AD patients. Interestingly, we found that liensinine and neferine could obviously reduce the accumulation of autophagosomes in worms (Fig. 10). These findings indicated that liensinine and neferine could enhance autophagy, while reducing the accumulation of autophagosomes due to abnormal fusion to maintain autophagy homeostasis.

Here, our results showed that liensinine and neferine could significantly inhibit Aβ and tau-induced toxicity by using APP695swe SH-SY5Y cells and *C. elegans* models. In addition, we also reported that liensinine and neferine have significant protective effects against Aβ-induced neuronal damage via improving oxidative stress tolerance and autophagy activity. Our findings provided a foundation for the use of liensinine and neferine to treat AD based on neuroprotective effects. Furtherly, we will focus on elucidate the structure activity relationship of bisbenzylisoquinoline alkaloids, and comprehensively evaluate their potential to treat AD by using mice model.

### Electronic supplementary material

Below is the link to the electronic supplementary material.

Additional file 1: **Table S1**. Liensinine and neferine improved cell viability. **Table S2**. Liensinine and neferine decreased intracellular ROS levels. **Table S3**. Liensinine and neferine delayed paralysis rate in C.elegans. **Table S4**. Liensinine and neferine enhanced chemotaxis behavior in C.elegans. **Table S5**. Liensinine and neferine enhanced 5-hydroxytryptamine sensitivity in C.elegans. **Table S6**. Liensinine and neferine improved motion ability in C.elegans. **Table S7**. Liensinine and neferine improved whipping rate in C.elegans. **Table S8**. Liensinine and neferine improve oxidative stress capacity in C.elegans. **Table S9**. Liensinine and neferine decreased ROS levels in C.elegans. **Table S10**. List of primers used in C. elegans. **Figure S1**. HPLC data of liensinine. **Figure S2**. NMR data of liensinine. **Figure S3**. HPLC data of neferine. **Figure S4**. NMR data of neferine. **Figure S5**. Gel image of marker. **Figure S6**. Gel image of LGG1-I/II-GFP.

## Data Availability

All data generated or analyzed during this study are included in this published article.
