# Supplementary material for: Liensinine and neferine exert neuroprotective effects via the autophagy pathway in transgenic Caenorhabditis elegans
Source: BMC Complement Med Ther. 2023 Oct 27;23:386. doi: 10.1186/s12906-023-04183-6 (PMC10612239; doi:10.1186/s12906-023-04183-6)
Supplement: Supplementary file 1 — Additional file 1: Table S1. Liensinine and neferine improved cell viability. Table S2. Liensinine and neferine decreased intracellular ROS levels. Table S3. Liensinine and neferine delayed paralysis rate in C.elegans. Table S4. Liensinine and neferine enhanced chemotaxis behavior in C.elegans. Table S5. Liensinine and neferine enhanced 5-hydroxytryptamine sensitivity in C.elegans. Table S6. Liensinine and neferine improved motion ability in C.elegans. Table S7. Liensinine and neferine improved whipping rate in C.elegans. Table S8. Liensinine and neferine improve oxidative stress capacity in C.elegans. Table S9. Liensinine and neferine decreased ROS levels in C.elegans. Table S10. List of primers used in C. elegans. Figure S1. HPLC data of liensinine. Figure S2. NMR data of liensinine. Figure S3. HPLC data of neferine. Figure S4. NMR data of neferine. Figure S5. Gel image of marker. Figure S6. Gel image of LGG1-I/II-GFP. [file 12906_2023_4183_MOESM1_ESM.docx]

**Supplementary data**

**Table S1.** Liensinine and neferine improved cell viability.

**Table S2.** Liensinine and neferine decreased intracellular ROS levels.

**Table S3.** Liensinine and neferine delayed paralysis rate in *C.elegans*.

**Table S4.** Liensinine and neferine enhanced chemotaxis behavior in *C.elegans*.

**Table S5.** Liensinine and neferine enhanced 5-hydroxytryptamine sensitivity in *C.elegans*.

**Table S6.** Liensinine and neferine improved motion ability in *C.elegans*.

**Table S7.** Liensinine and neferine improved whipping rate in *C.elegans*.

**Table S8.** Liensinine and neferine improve oxidative stress capacity in *C.elegans*.

**Table S9.** Liensinine and neferine decreased ROS levels in *C.elegans*.

**Table S10.** List of primers used in *C. elegans.*

**Figure S1.** HPLC data of liensinine.

**Figure S2.** NMR data of liensinine.

**Figure S3.** HPLC data of neferine.

**Figure S4.** NMR data of neferine.

**Figure S5.** Gel image of marker.

**Figure S6.** Gel image of LGG1-Ⅰ/Ⅱ-GFP.

**Table S1.** Liensinine and neferine improved cell viability.

| Treatment | SH-SY5Y | APPswe | 5μM Liensinine | 10μM Liensinine | 20μM Liensinine | 5μM  Neferine | 10μM  Neferine | 20μM  Neferine |
| --- | --- | --- | --- | --- | --- | --- | --- | --- |
| Cell viability (%) | 100 | 71.95±1.20 | 79.30±2.55 | 85.55±1.20 | 80.00±3.25 | 78.55±0.64 | 89.15±0.78 | 83.75±5.30 |
| *p* value | - | 0.0009 | - | 0.0077 | - | 0.0099 | 0.0034 | - |

**Table S2.** Liensinine and neferine decreased intracellular ROS levels.

| Treatment | Concentration (μM) | ROS (%) | *p* value |
| --- | --- | --- | --- |
| Control | 0 | 100 | - |
| Liensinine  Neferine | 10  10 | 84.69±3.26  79.63±2.66 | 0.0012  0.0002 |

**Table S3.** Liensinine and neferine delayed paralysis rate in *C.elegans*.

| Treatment | Control | 50μM Liensinine | 100μM Liensinine | 200μM Liensinine | 50μM  Neferine | 100μM  Neferine | 200μM  Neferine |
| --- | --- | --- | --- | --- | --- | --- | --- |
| PT_50_(h) | 2.62±0.10 | 3.00±0.03 | 3.79±0.24 | 2.98±0.20 | 3.12±0.14 | 4.20±0.05 | 3.49±0.14 |
| PT_50_ extension percentage | 14.50% | 44.66% | 13.74% | 19.08% | 60.31% | 33.21% |  |
| *p* value | - | 0.0336 | 0.0243 | - | - | 0.0023 | 0.0175 |

**Table S4.** Liensinine and neferine enhanced chemotaxis behavior in C.*elegans*.

| Treatment | Concentration (μM) | CI | *p* value |
| --- | --- | --- | --- |
| Control | 0 | -0.13±0.02 | - |
| Liensinine  Neferine | 100  100 | 0.16±0.02  0.18±0.01 | 0.005  0.003 |

**Table S5.** Liensinine and neferine enhanced 5-hydroxytryptamine sensitivity in C.elegans.

| Treatment | Concentration (μM) | Proportion of active nematodes | *p* value |
| --- | --- | --- | --- |
| Control | 0 | 24.84±3.06 | - |
| Liensinine  Neferine | 100  100 | 46.20±2.55  56.95±1.91 | 0.017  0.008 |

**Table S6.** Liensinine and neferine improved motion ability in *C.elegans*.

| Treatment | Concentration (μM) | Average moving distance within 10 seconds (μM) | *p* value |
| --- | --- | --- | --- |
| Control | 0 | 71.87±3.38 | - |
| Liensinine  Neferien | 100  100 | 85.93±0.40  87.85±0.57 | 0.002  0.0013 |

**Table S7.** Liensinine and neferine improved whipping rate in *C.elegans*.

| Treatment | Concentration (μM) | Whipping rate | *p* value |
| --- | --- | --- | --- |
| Control | 0 | 3.77±0.61 | - |
| Liensinine  Neferien | 100  100 | 8.73±0.88  9.41±0.30 | 0.0013  0.0001 |

**Table S8.** Liensinine and neferine improve oxidative stress capacity in *C.elegans*.

| Treatment | Concentration (μM) | Average survival time (h) | Elongation rate (%) | *p* value |
| --- | --- | --- | --- | --- |
| Control | 0 | 37.47±0.04 | - | - |
| Liensinine  Neferine | 100  100 | 40.07±0.85  42.49±0.32 | 6.94%  13.40% | 0.049  0.033 |

**Table S9.** Liensinine and neferine decreased ROS levels in *C.elegans*.

| Treatment | Concentration (μM) | Relative fluorescence percentage (%) | *p* value |
| --- | --- | --- | --- |
| Control | 0 | 100 | - |
| Liensinine  Neferine | 100  100 | 73.77±6.90  67.13±3.71 | 0.003  0.0001 |

**Table S10.** List of primers used in *C. elegans.*

| **Gene** | Forward primer | Reverse primer |
| --- | --- | --- |
| *actin-1* | TCGGTATGGGACAGAAGGAC | CATCCCAGTTGGTGACGATA |
| *unc-51* | GTGCTCTCCGAATCTACGGG | GGTATGCACTTGGACCTGCT |
| *lgg-1* | GCCGAAGGAGACAAGATCCG | GGTCCTGGTAGAGTTGTCCC |
| *atg-9* | ATCTAAAACACGAAATCGAGCG | CTCGTGATGTTTGTACTCCTCT |
| *pha-4*  *ced-9* | CACGCAAGCACAGATGACAC  GGGAAGAGCCAAGGCTTGAT | ATGGCAGGAGGATCGCAAAA  CATGAAGTCGTCCCAGCTCC |
| *sod-3* | CAATTGCTCTCCAACCAGCG | ACCGAAGTCGCGCTTAATAG |
| *gst-4* | ACCAGCCCGTGATGATTTCT | ATCCTTTCTTGTTGCCACGT |


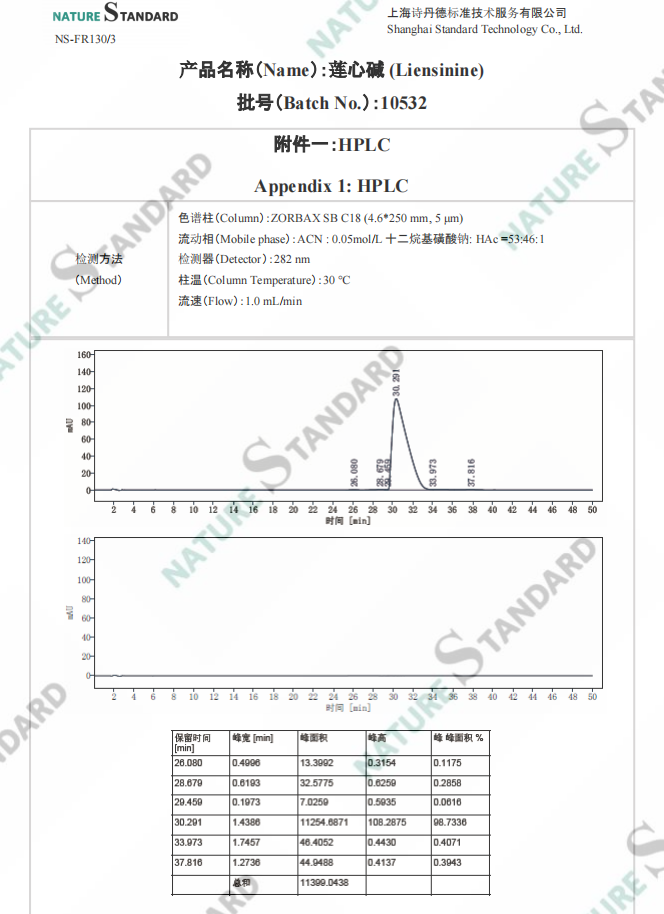


**Figure S1.** HPLC data of liensinine.


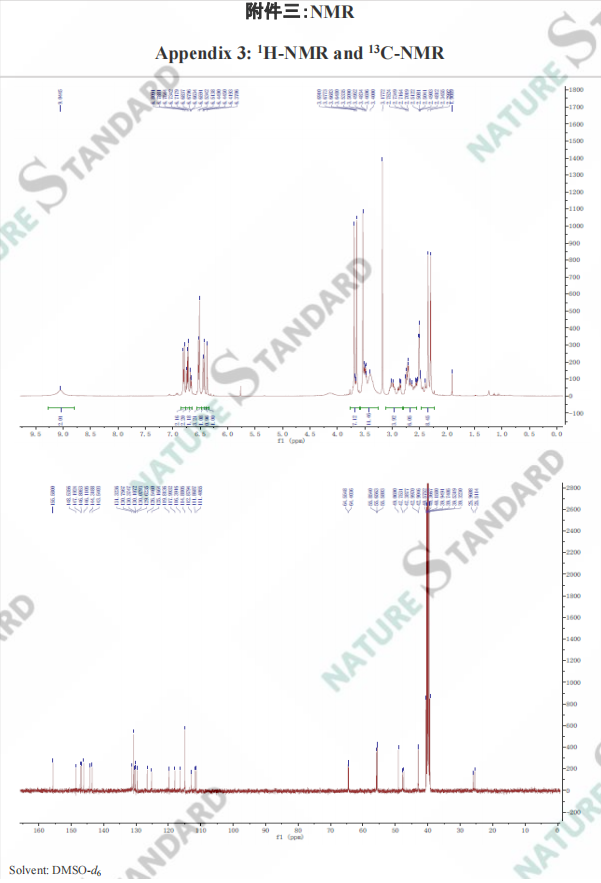


**Figure S2.** NMR data of liensinine.


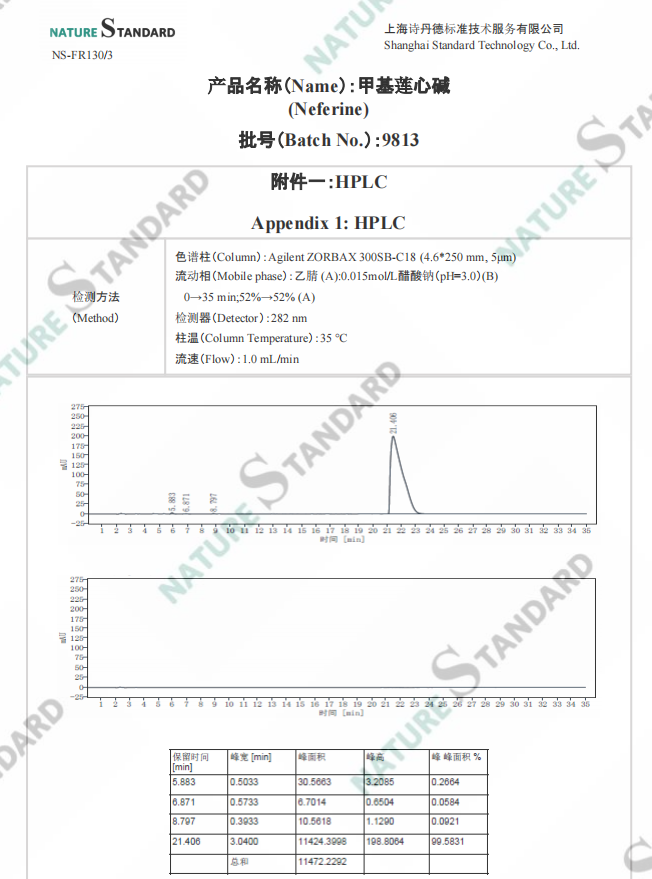


**Figure S3.** HPLC data of neferine.


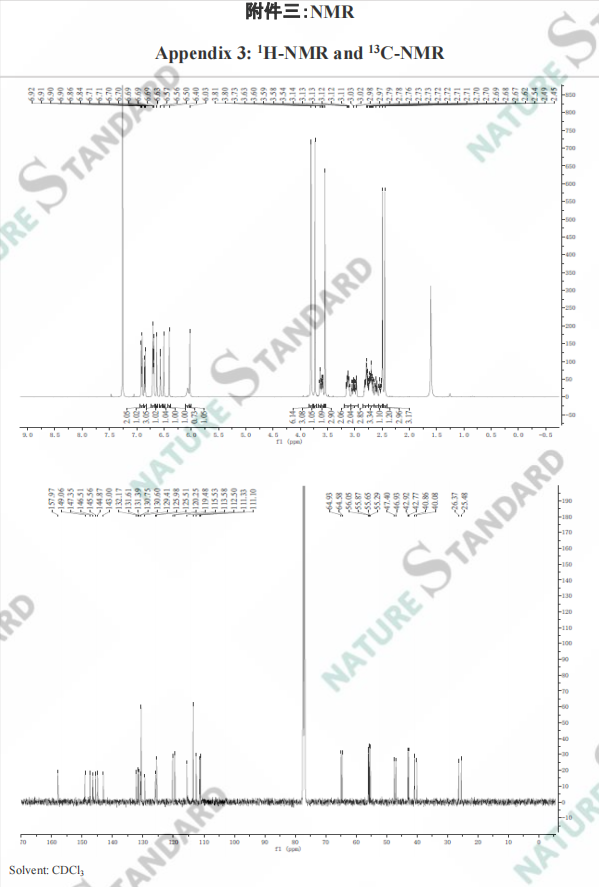


**Figure S4.** NMR data of neferine.


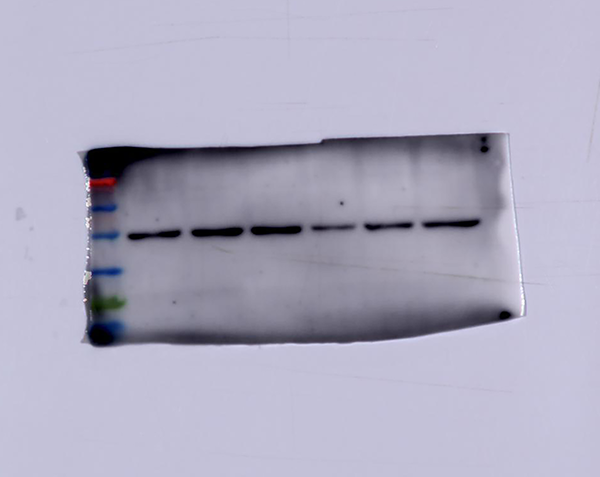


**Figure S5.** Gel image of marker.


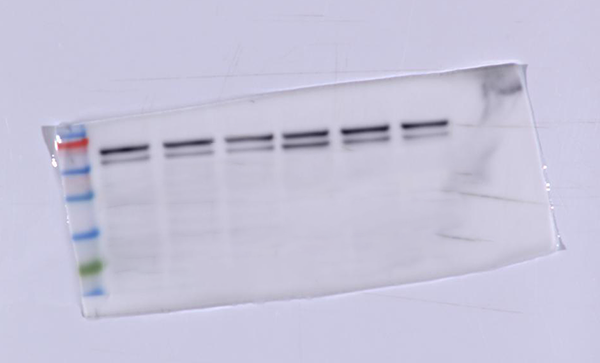


**Figure S6.** Gel image of LGG1-Ⅰ/Ⅱ-GFP.
